# Editorial: LGBT Inclusion in Schools

**DOI:** 10.3389/fsoc.2022.904357

**Published:** 2022-04-26

**Authors:** Jonathan Glazzard, Mark Vicars

**Affiliations:** ^1^Faculty of Education, Edge Hill University, Ormskirk, United Kingdom; ^2^College of Arts and Education, Victoria University, Melbourne, VIC, Australia

**Keywords:** LGBTQ+, inclusion, schools, education, queer

The likelihood that your acts of resistance cannot stop the injustice does not exempt you from acting in what you sincerely and reflectively hold to be the best interests of your community.

Susan Sontag (2007).

In proposing this Research Topic, we were interested in hearing accounts from practitioners and scholars who are involved in studying the narratives associated with LGBTQ+ experiences across a range of educational, community and cultural domains. Research continues to suggest that queer young people experience higher rates of depression, anxiety and self-harm compared to their heterosexual peers (Goldbach and Gibbs, 2017). Evidence also indicates that they are more likely to attempt suicide (Marshal et al., 2011) and experience eating disorders (Austin et al., 2013). We hoped that the Research Topic would engage with the multitude of stories that represent the diversity of LGBTQ+ lives as lived and the authors of the papers are globally situated and united around “flipping the (normative) script to survive” (Giroux, 2015).

The authors in this Research Topic narrate a contemporary account of the social realities as experienced by LGBTQ+ individuals and their allies, and in doing so rewrite the dominant narratives so often expressed in and by minority stress theory (Meyer, 2003) which considers how stress can affect the mental health outcomes in individuals with a minority status. The Research Topic engages with a critical reorientation to the field to elucidate how minority status can expose Queer individuals and their allies to three stressors:

- General stressors: These are stressors which result from environmental circumstances. They could include family factors such as parental conflict, parental separation, abuse and neglect and community-related factors such as social deprivation.
- Distal stressors: These are stressors which arise from the experience of prejudice, discrimination and violence.
- Proximal stressors: These are stressors which arise from the expectations of rejection, prejudice, discrimination and violence. That is, an individual with a minority status does not have to actually experience distal stressors but the anticipation that they might encounter these stressors in different social and environmental contexts can result in psychological distress, concealment and internalized homophobia. For example, they may anticipate that they will encounter prejudice if they disclose their sexual or gender identity. This can lead to students concealing their identities, which can result in internalized stigma.

It is important to acknowledge that while much progress has been made for LGBTQ+ inclusion to become widely recognized and accepted as the “norm” that there still remains, for many young Queer, Non-Binary and LGBT individuals, negative social and cultural experiences when disclosing their identities (distal stressors) which may increase expectations of further rejection (proximal stressors) in different contexts (Goldbach and Gibbs, 2017). Additionally, concealing one's identity due to fear of rejection (proximal stressor) can reduce the likelihood of experiencing prejudice, discrimination and violence (distal stressor) (Goldbach and Gibbs, 2017). Thus, the stressors are inter-related and bi-directional. Meyer's theory suggests that the stressors can be moderated by social support systems that are specifically established to foster both group solidarity and positively affirm minority identities. Many schools now provide “safe spaces” for students who identify as LGBTQ+ to meet informally. These groups enable students to provide one another with mutual support and advice. Some groups also adopt a proactive approach to LGBTQ+ inclusion within the school by developing initiatives to embed LGBTQ+ inclusion into the curriculum and the environment. Providing opportunities for queer students to meet as a group can enhance social connectivity, reduce internalized stigma and increase resilience. However, there is also a risk that separating out one group of students in this way can also result in internal exclusion though the creation of an “othered” group. One way of addressing this is to allow membership of the group to heterosexual allies who are deeply committed to LGBTQ+ inclusion.

This Research Topic aims to extend existing scholarship in the field in the tradition of Waller (1932), Lawrence-Lightfoot (1983) and Cutuly (1993) and draws on quotidian portraits that account for the multiplicity of ways in which LGBTQ+ individuals, including students, teachers and allies, juggle the complexity of competing personal and professional demands whilst facing moral and ethical conflicts within their institutions. Darder (2011, p. 238) has noted how

[Queer} bodies are … restricted, alienated, and domesticated…, [and] are often under enormous pressure to follow strict policies and procedures for classroom conduct.

Adolescence is also a critical time during which young people explore their sexual and gender identities and research indicates that stigmatizing experiences during adolescence can reduce academic achievement and result in negative outcomes later in life (Radkowsky and Siegel, 1997). Prior to disclosure, LGBTQ+ young people may have experienced psychological distress from internalized stigma, particularly as they are coming to terms with their sexual or gender identities. The anticipation that their disclosure might be met with hostility or disgust can result in fear of disclosure (proximal stressor) and concealment of identities.

The process of “coming out” can result in exposure to both proximal and distal stressors for young people and the authors in this Research Topic address how individual and personal experiences (can critically) orient and provide valuable insight into effective strategies for coping (or not) with professional/ personal obstacles and adversity and stigma. The authors, in narrating the pedagogy of the “abjected,” offer up another lens, a different worldview which is a significant critical presence of interruption in the contemporary performativity of heteropatriarchal and cis gender normativitites. Goodson and Gill (2011) have noted that when narratives of (normative) performativity are entrenched, disruption and provocation are required and in speaking back a truth to power. In this Research Topic, we have endeavored to (re)present a kaleidoscopic insight into the social and cultural landscapes in which we live as Gay, Queer, Trans, Non-Binary and Two Spirit people to challenge the tidal cacophony of those who would rather have us remain silent.

## Author Contributions

All authors listed have made a substantial, direct, and intellectual contribution to the work and approved it for publication.

## Conflict of Interest

The authors declare that the research was conducted in the absence of any commercial or financial relationships that could be construed as a potential conflict of interest.

## Publisher's Note

All claims expressed in this article are solely those of the authors and do not necessarily represent those of their affiliated organizations, or those of the publisher, the editors and the reviewers. Any product that may be evaluated in this article, or claim that may be made by its manufacturer, is not guaranteed or endorsed by the publisher.
